# Traumatic iatrogenic chylothorax after external electrical cardioversion

**DOI:** 10.1002/rcr2.695

**Published:** 2020-12-03

**Authors:** Maria Ralli, Tobias Justesen, Susanne C. Frevert, Anders N. Ø. Schultz

**Affiliations:** ^1^ Department of Internal Medicine Hospital Southern Jutland Sonderborg Denmark; ^2^ Department of Diagnostic Radiology Rigshospitalet Copenhagen Denmark

**Keywords:** Chylothorax, embolization, external electrical cardioversion, lymphangiography, trauma

## Abstract

Chylothorax, an uncommon cause of pleural effusion, results from the accumulation of lymph in the pleural space due to damage or obstruction of the thoracic duct. Chylothorax can be due to several aetiologies, many of which are rare, and it is often a diagnostic challenge to identify the cause. This case report refers to a patient with rapid recurrent episodes of bilateral pleural chylothorax due to damage of the thoracic duct after external electrical cardioversion treatment. The diagnosis took place by the method of exclusion, when all known causes of chylothorax, both non‐traumatic and traumatic, were ruled out. A review of the literature on chylothorax was performed using PubMed to assess the different aetiologies, investigations, and treatments usually performed. Chylothorax is usually secondary to malignancy, trauma, congenital diseases, and infections. However, even non‐invasive thoracic procedures, such as the one described in our case report, can be the cause.

## Graphical abstract

This case report refers to a patient with rapid recurrent episodes of bilateral pleural chylothorax due to damage of the thoracic duct after external electrical cardioversion (EEC) treatment. The diagnosis took place by the method of exclusion, when all known causes of chylothorax, both non‐traumatic and traumatic, were ruled out. To the authors' knowledge, no case of traumatic chylothorax after EEC has been reported in the literature.

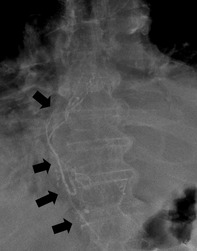

## Introduction

Chylothorax is a rare and serious manifestation of pleural effusion, which can occur due to non‐traumatic or traumatic damage to the thoracic duct or any of its tributaries [1]. More than half of the traumatic cases are iatrogenic in nature, mainly caused by surgery or invasive procedures [2]. We report a patient who developed chylothorax following an external electrical cardioversion (EEC), performed to treat atrial fibrillation. To the authors' knowledge, chylothorax as a complication to EEC has not previously been reported.

## Case Report

The patient was a 79‐year‐old man, who presented to our emergency department with acute dyspnoea. The patient had a three‐year history of paroxysmal atrial fibrillation, which was conservatively treated, because it spontaneously restored to sinus rhythm. He was diagnosed with chronic obstructive pulmonary disease (Global initiative for chronic Obstructive Lung Disease (GOLD) group B) and mild pulmonary hypertension. Furthermore, the medical history included depression, inactive multinodular goitre, and previous colon polypectomy.

In the previous month, he had experienced palpitations and progressive dyspnoea diagnosed as atrial fibrillation, and was treated with bisoprolol, digoxin, and apixaban. However, this was without sufficient clinical response, so he was subsequently treated with EEC (biphasic, 120 J), which restored him to sinus rhythm and relieved him of the dyspnoea and the palpitations.

The procedure was successfully repeated 11 days later, due to a new episode of dyspnoea and palpitations, which again was diagnosed as atrial fibrillation. Five days after the second EEC, the man presented to our emergency department with acute dyspnoea. At the time of admission, ultrasound revealed significant pleural effusion on the right hemithorax. Thoracentesis was performed on the right hemithorax and 2 L of milky fluid was collected. The pleural fluid was sent to standard laboratory analysis, which included complete blood count and white blood cell differential, serum glucose, total protein, and lactate dehydrogenase. In addition, the analysis involved cholesterol and triglyceride measurement because fluid appearance raised suspicions for chylothorax. Pleural fluid was transudate with a high concentration of triglycerides (6.11 mmol/L) and a low level of cholesterol (1.3 mmol/L). The pleural effusion was classified as chylothorax and further research was undertaken to discover the underlying cause.

The patient underwent clinical and laboratory testing, including microbiological and cytological analyses of the pleural fluid as well as computed tomography (CT) and positron emission tomography CT of the chest, abdomen, and pelvis; spirometry; 6‐min waking test; and echocardiography. He was tested for connective tissue disorders, sarcoidosis, tuberculosis, lymphangioleiomyomatosis, and amyloidosis, all of which were ruled out. Malignancy, including lymphoma and myeloma, and benign tumours were also excluded. The patient did not have a history of thoracic radiation. There were no signs of infectious disease. Liver cirrhosis, heart failure, nephrotic syndrome, congenital or idiopathic disorders of the lymphatic system, and lymphatic conduction disorders were excluded. Hence, any known cause to non‐traumatic chylothorax due to malignant and non‐malignant conditions was ruled out after thorough investigation.

Simultaneously, traumatic causes were investigated. There was no medical history of previous surgical procedures in the area of the thoracic duct or nearby structures, thus surgical traumatic causes could be ruled out. The only predisposing factor and thus the only reasonable explanation for our patient's pathology was traumatic chylothorax after EEC.

During the whole examination process, the patient suffered from rapid recurrent episodes of bilateral pleural effusions all of which were classified as chylothorax. The patient was symptomatically treated with thoracenteses on both hemithoraces.

In addition to thoracenteses, the patient was treated conservatively with dietary modification. At first, he followed an oral low‐fat diet (<10 g fat/day) with no results. Second, he followed total parenteral low‐fat diet, similarly without results. The low‐fat diet was provided and controlled by nutritionist in both cases. Finally, the patient underwent talc pleurodesis, preceded by thoracentesis on the right hemithorax. However, post‐procedure chest X‐ray (CXR) revealed recurrent pleural effusion.

**Figure 1:**
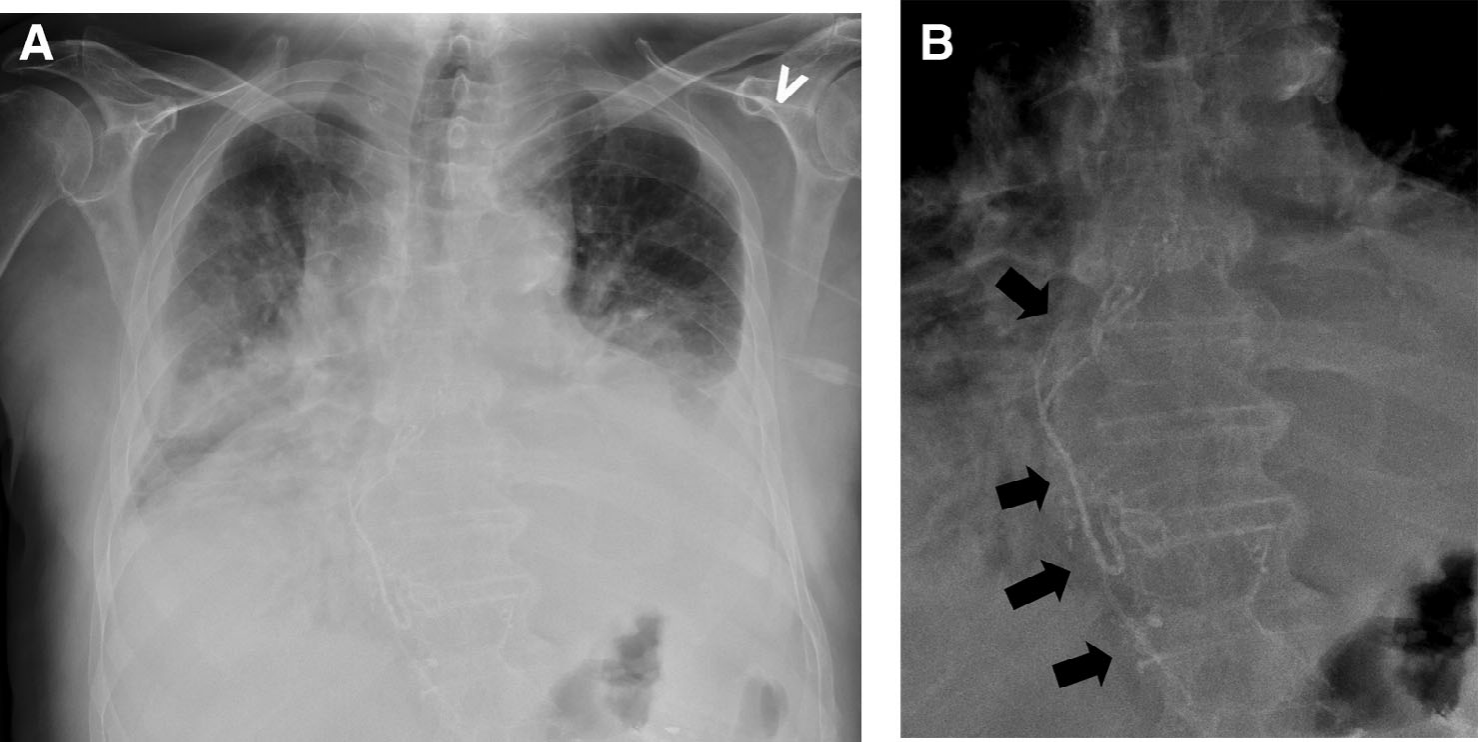
(A) Chest X‐ray from the lymphangiography. (B) Enlarged version of (A), with arrows pointing to the embolized thoracic duct.

Thus, our next choice was lymphangiographic presentation of the thoracic duct with embolization. Lymphangiography is a contrast‐enhanced study of the lymphatic system that can delineate thoracic duct anatomy and identify a potential site of chyle leak. Lymphangiography can identify areas of chyle leakage and lymphangiectasia in the majority of patients. In our case, a lymph node in the groin was punctured and Lipiodol was injected until the cisterna chyli was visualized. The cisterna chyli was then punctured and as it was impossible to advance the guide more than a few centimetres into the lymphatic duct, the affected thoracic duct was then embolized using Histoacryl/Lipiodol. No coils were used. Figure 1 shows the lymphangiographic presentation of the thoracic duct filled with Histoacryl/Lipiodol (Braun Medical A/S, Denmark).

After the lymphangiography and embolization of the thoracic duct, the patient underwent a new CXR, which, for the first time since the initial diagnosis, did not reveal pleural effusion.

## Discussion

This case report refers to a patient with rapid recurrent episodes of bilateral pleural chylothorax due to damage of the thoracic duct after EEC treatment. To the authors' knowledge, no case of traumatic chylothorax after EEC has been reported in the literature. However, in our case patient, all the known causes of traumatic and non‐traumatic chylothorax were excluded after comprehensive evaluation and thorough investigation, and lymphangiographic presentation of the thoracic duct with embolization led to therapeutic results. Thus, it was concluded that trauma of the thoracic duct following EEC was the underlying aetiology for the patient's chylothorax.

Finding the underlying aetiology of chylothorax can be a diagnostic challenge as our case illustrates, in part due to the great number of aetiologies including both traumatic and non‐traumatic causes. Chylothorax due to trauma is often readily explained, as the majority of cases are due to surgical procedures in the area of the thoracic duct or nearby structures [2, 3], while non‐traumatic causes are much more varied and subdivided into malignant or non‐malignant conditions, with malignancy as the leading cause [4]. Another approximately 6% of all chylothorax cases are classified as idiopathic [2]. The case patient posed a further diagnostic challenge, as chylothorax due to EEC had not been previously reported, which meant that all other known causes had to be ruled out before we could reach that conclusion.

The patient was, in both cases, treated with a biphasic shock at an energy level of 120 J, with the electrodes anterior–posterior positioned. At the level of tracheal bifurcation, the thoracic duct is anteriorly related to the oesophagus and the left atrium. The electroanatomical features of the anterior–posterior position of electrodes include both atria in the shock field, in fact especially the left atrium, which means that the current reaching the myocardium is likely to reach the thoracic duct as well [5]. Although complications to EEC are minimal, they do include pulmonary oedema, myocardial necrosis, and skin burn among others [6]. In light of the anatomical relations of the thoracic duct and the known complications of EEC, it is plausible that the energy delivered during the EEC would be sufficient to cause damage to the thoracic duct leading to chylothorax.

In conclusion, chylothorax is a rare condition with multiple aetiologies. Patients with an established diagnosis of chylothorax should undergo additional clinical re‐evaluation and laboratory testing for aetiologies that may have been missed during the initial evaluation. In case of no evidence‐based explanation for chylothorax, lymphangiography can be performed. This case report highlights the diagnostic challenge of finding the aetiology of chylothorax and the fact that even minimal non‐invasive thoracic procedures, such as EEC, can be the cause.

### Disclosure Statement

Appropriate written informed consent was obtained for publication of this case report and accompanying images.
